# Cenobamate reduces epileptiform activity in the *ex vivo* F98 rat glioma model

**DOI:** 10.3389/fnins.2025.1629259

**Published:** 2025-08-05

**Authors:** Ferdinand Forberger, Fabiana Santana Kragelund, Katrin Porath, Rüdiger Köhling, Timo Kirschstein, Falko Lange

**Affiliations:** ^1^Oscar Langendorff Institute of Physiology, University of Rostock, Rostock, Germany; ^2^Center for Transdisciplinary Neurosciences Rostock, University of Rostock, Rostock, Germany

**Keywords:** cenobamate, glioma, epilepsy, electrophysiology, deep learning

## Abstract

**Introduction:**

Epileptic seizures are a common clinical sign in patients suffering from high-grade glioma. In addition to therapeutic interventions aiming to prolong the remaining lifespan, maintaining quality of life is a cornerstone of current treatment concepts. Consequently, anticonvulsants are frequently applied to keep seizures at bay, but drug resistance is still a challenge. There is a need for new anticonvulsants to address this issue. Therefore, for the first time, we evaluated the efficacy of cenobamate, a novel anticonvulsant shown to inhibit persistent sodium currents and modulate GABA_A_ receptor function, in a preclinical model of glioma-associated epilepsy. In our study, we used cortical slices from naive Fischer rats and animals with orthotopically implanted F98 tumors.

**Methods:**

To study the effect of cenobamate (60 and 120 μmol/L), we recorded local field potentials and evoked spontaneous network deflections using an acute disinhibition solution. To analyze seizure-like events (SLEs), we employed a deep learning approach, further supported by power spectral density (PSD) analysis.

**Results:**

Cenobamate attenuated the proportion of recording time occupied by SLEs, mainly by reducing their duration in slices of both sham-operated and F98 tumor-bearing animals. Additionally, the spike load within SLEs was diminished by the anticonvulsant. The PSD analysis confirmed the reduction of spike frequencies abundant in seizure-like events.

**Conclusion:**

Our data show that cenobamate effectively reduced the epileptic phenotype in glioma-bearing brain slices. We hence suggest that cenobamate may effectively contribute to seizure control in tumor-associated epilepsy.

## 1 Introduction

Epileptic seizures are a common symptom of glioma. In low-grade gliomas, 70–90% of the patients suffer from tumor-related epilepsy. At least half of patients diagnosed with high-grade glioma/astrocytoma exhibit seizures (Kerkhof and Vecht, 2013; van Breemen et al., 2007). Seizure control is sought through an interdisciplinary approach including radiochemotherapy, surgery, and administration of anticonvulsants (Weller et al., 2024). Medical treatment diminishes the generation of excitatory postsynaptic potentials and action potentials or, conversely, enhances inhibitory mechanisms in the central nervous system (Armstrong et al., 2016; Löscher and Klein, 2021). However, drug-resistant epilepsy was reported in up to 30% of patients suffering from glioma-associated seizures (Correia et al., 2021; Goldstein and Feyissa, 2018; Stritzelberger et al., 2024). Therefore, establishing new pharmaceutics as anticonvulsants to achieve seizure freedom is highly desirable. Cenobamate (CNB) is a novel anticonvulsant (Błaszczyk et al., 2024; Kwan and Brodie, 2025) that may help to overcome drug resistance in glioma-associated epilepsy.

Irrespectively of the cause of epilepsy, a multicentric study by Krauss et al. in 2020 on patients with drug-resistant epilepsy showed a dose-dependent reduction of seizure frequency by orally administered adjunctive CNB (Krauss et al., 2020). Another phase II study also on patients with drug-resistant epilepsy confirmed results regarding seizure frequency (Chung et al., 2020). Later studies under real-world conditions underscore the beneficial effect of CNB in various cohorts of patients suffering from drug-resistant epilepsy (Loushy et al., 2025; Rheims et al., 2025; Roberti et al., 2024; Rodríguez-Uranga et al., 2024).

Based on current knowledge, CNB exerts its anticonvulsant effects through two distinct mechanisms of action. First, Nakamura et al. (2019) showed that CNB is an allosteric antagonist of voltage-gated sodium channels (Nakamura et al., 2019). The modulation primarily affects persistent Na^+^ currents and has minimal effect on transient Na^+^ currents. Physiologically, the window current via voltage-gated sodium channels (Müller et al., 2024) is reduced by CNB, resulting in a hyperpolarization of the cell transmembrane potential, which increases the threshold for generating action potentials and thereby reduces their frequency. No effects were observed on excitatory currents mediated by ionotropic glutamate receptors or on voltage-gated calcium and potassium currents (Nakamura et al., 2019).

A second, different mechanism was identified to be based on an increased GABA_A_ receptor-mediated transmission (Sharma et al., 2020). While CNB alone failed to induce receptor opening, it acts as an allosteric agonist for GABA-induced outward currents in a dose-dependent manner. While at low GABA concentrations, CNB facilitated the Cl^−^ current; at high concentrations of the transmitter, no additional effect by the anticonvulsant was detected. The authors concluded that CNB modulates extra-synaptic tonic GABA_A_ receptors and that the impact at the synapse via phasic GABA-mediated currents may play a minor role only.

So far, CNB has not been established in the treatment of glioma-related epilepsy, and neither preclinical data nor studies involving humans have been reported. Albeit the acute brain slice preparation may only partly reflect drug resistance *in vivo*, it offers the opportunity for an in-depth analysis of pharmacological effects, in particular when deep learning algorithms for rapid data processing are concerned. Here, we used the F98 glioma model in Fischer 344 rats and hypothesized that CNB would attenuate epileptic network activity. To this end, we evoked spontaneous epileptic network activity in the absence or presence of CNB and obtained robust and comparative CNB effects in both experimental groups.

## 2 Materials and methods

### 2.1 Cell culture and cenobamate preparation

F98 glioma cells were purchased from the American Type Culture Collection (ATCC). The cells were cultured in Dulbecco’s Modified Eagle Medium: Nutrient Mixture F-12 (DMEM/F12; from PAN Biotech, Aidenbach, Germany) with 10% fetal calf serum (FCS, Bio&SELL, Feucht, Germany). Cell-culturing was done at 37°C in a 5% CO_2_ humidified atmosphere. At constant intervals, MycoSPY^®^-PCR Mycoplasma Test Kit (Biontex, Munich, Germany) was used to test cell culture supernatants for mycoplasma contamination. During the project period, no contamination with mycoplasmas was detected. Cenobamate tablets (Ontozry, Angelini Pharma, Rome, Italy) were crushed thoroughly and dissolved in 98% ethanol. The suspension was centrifuged and filtered (40 μm) to yield a 120 mM stock solution. In our experiments, the concentration of ethanol in artificial cerebrospinal fluid (aCSF) was 5‰ (v/v).

### 2.2 Animal tumor model and stereotactic glioma implantations

All procedures were conducted according to national and international guidelines on the ethical use of animals (European Council Directive 86/609/EEC, approval of local authority LALLF M-V/TSD/7221.3–1-020/20). All efforts were made to minimize animal suffering and to reduce the number of animals used. The Fischer 344 rats were housed under environmentally controlled conditions (12 h light/dark cycles, lights switched on from 6 a.m. to 6 p.m., and 40–60% relative humidity). For the present study, a total of 29 Fischer 344 rats were included.

To imitate human brain cancer, F98 glioma cells were stereotactically implanted in the sensorimotor neocortex of male Fischer 344 rats (Charles River, Sulzfeld, Germany) at the age of 8–10 weeks as previously described (Lange et al., 2020). Briefly, the animals were anesthetized with ketamine (100 mg/kg i.p.) and xylazine (10 mg/kg i.p.), and the head was fixed in a stereotactic frame (Narishige, Tokyo, Japan). Following a scalp incision, the skull was freed from extracranial muscles, and a hole of 0.7 mm diameter was manually drilled into the skull in the left parasagittal position (relative to bregma: 1.8 mm posterior, 2.5 mm left, 2 mm deep). Next, F98 cells from a subconfluent-growing culture were prepared for injection with 1 × 10^5^ cells/μL phosphate-buffered saline (PBS; injection rate: 0.5 μL/min; total volume: 10 μL) using a Hamilton syringe (Model 701 N SYR; Hamilton, Reno, Nevada, United States). After injection of the cell suspension, the drill hole was covered with Heliobond (Ivoclar Vivadent, Schaan, Lichtenstein), and the scalp was closed with a suture. Sham-operated animals underwent the same procedure with 10 μL PBS instead of F98 cell suspension. Ten to twelve days after surgery, Fischer rats were sacrificed and subjected to electrophysiological investigations. Based on previous investigations, we know that glioma-bearing Fischer rats exhibit interictal spikes and seizures (Lange et al., 2020; Bouckaert et al., 2020).

### 2.3 Neocortical slice preparations

For electrophysiological recordings, Fischer 344 rats were deeply anesthetized with diethyl ether (Thermo Fisher Scientific, Waltham, MA, United States) and subsequently decapitated. The brain was rapidly extracted and immediately transferred into a chilled, oxygenated dissection solution (95% O₂/5% CO₂) containing (in mmol/L) 87 NaCl, 25 NaHCO₃, 2.5 KCl, 1.25 NaH₂PO₄, 0.5 CaCl₂, 7 MgCl₂, 10 D-glucose, and 75 sucrose, adjusted to pH 7.4 with an osmolarity of 320–330 mosmol/L. After removing the cerebellum, coronal brain slices were prepared using a vibratome (LEICA VT1200, Wetzlar, Germany). 500-μm slices were prepared in a chilled solution continuously bubbled with carbogen. The slices were then transferred to a submerged storage chamber and maintained in aCSF equilibrated with carbogen. The aCSF was composed of (in mmol/L) 124 NaCl, 26 NaHCO₃, 3 KCl, 1.25 NaH₂PO₄, 2.5 CaCl₂, 1.5 MgCl₂, and 10 D-glucose, adjusted to pH 7.4 with an osmolarity of 304–312 mosmol/L. The slices were stored for one to 6 h before being transferred to the interface chamber for electrophysiological investigation.

### 2.4 Electrophysiological recordings

The coronal slices were transferred into an interface chamber (BSC-HT, Harvard Apparatus, Holliston, MA, United States) and continuously perfused with aCSF bubbled with carbogen. The chamber temperature was kept constant at 31 ± 1°C (TC-10, npi electronic GmbH, Tamm, Germany). Under visual control, electrodes were positioned within a rim of 1 mm around the glioma, and field potentials were recorded using conventional aCSF-filled glass micropipette electrodes (Ag/AgCl, with a resistance of approximately 2–5 MΩ). The analog recordings were amplified, low-pass filtered at 30 Hz (EXT-10-2F, npi electronic GmbH), and digitized using a Micro1401 analog-to-digital converter (Cambridge Electronic Design, Cambridge, United Kingdom). Signal 2.16 (Cambridge Electronic Design) was used to save recordings with data sampled at 2,000 Hz. After being introduced into the measurement chamber, slices were perfused using aCSF. Since acute slices do not show spontaneous activity unless they are exposed to disinhibiting conditions, aCSF was modified to evoke spontaneous network disinhibitions. Two different protocols were tested: (I) aCSF with 8 mM KCl and 0 mM MgCl_2_ and (II) aCSF containing 8 mM KCl, 0 mM MgCl_2_ and 50 μmol/L 4-aminopyridine (4-AP; Tocris, Bristol, United Kingdom). The solutions were adjusted to a pH of 7.4 with an osmolarity of 304–312 mosmol/L. Robust occurrence of seizure-like events (SLEs) was typically reached by minute 80. Data recorded from 100 to 140 min were used for further analysis. We defined SLEs as clearly distinguishable, repetitive spiking events in local field potential (LFP) activity. These events had to exhibit similar morphology, appear in an almost rhythmic fashion for an extended period, last at least 0.2 s, and had to be separated by a minimum of 0.1 s from preceding and subsequent events.

**Figure 1:**
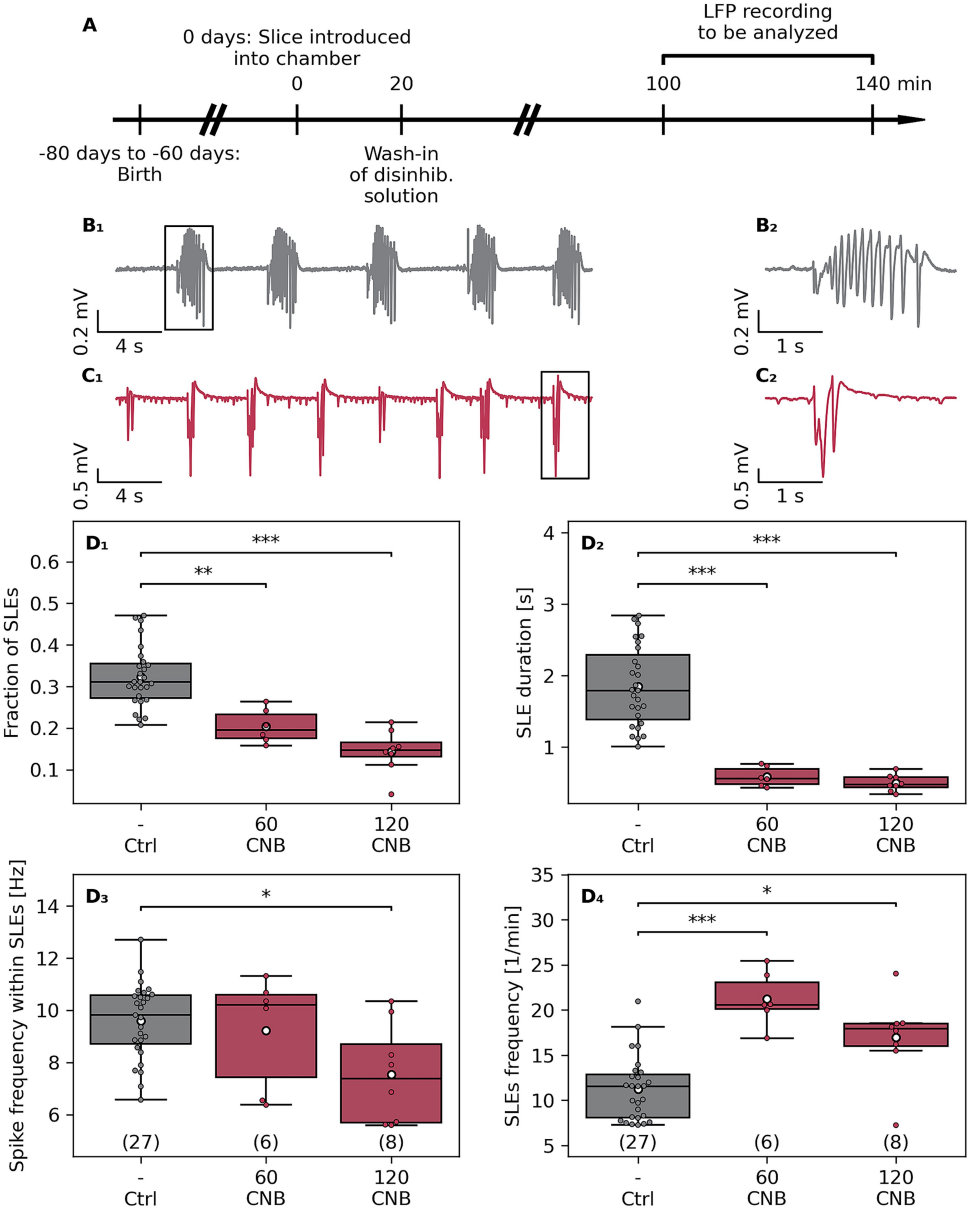
Effects of cenobamate on network excitability of naive murine slices. **(A)** Male Fischer 344 rats at the age of 60–80 days were sacrificed, and subsequently, coronal brain slices were subjected to electrophysiological investigation. **(B**_**1**_**)** Sample traces of local field potential (LFP) recordings of a vehicle-treated (Ctrl, **B**_**1**_) and cenobamate-exposed (60–120 μmol/L CNB, **C1**) slices. **(B**_**2**_**)** respectively **(C**_**2**_**)** represent magnified views of a seizure-like event (SLE) marked with black boxes in **(B1,C1)**. **(D**_**1–4**_**)** Quantitative analysis of SLE characteristics represented as boxplots. The number of coronal slices is given in brackets [Ctrl: 27 slices (9 rats), 60 μmol/L CNB: 6 slices (3 rats), and 120 μmol/L CNB: 8 slices (4 rats)]. Individual data points are presented as full-colored dots. Median is shown as a black-colored line, and the mean is illustrated as a white-colored circle; **(D**_**1**_**,**_**2**_**,**_**4**_**)** **p < 0.01,***p < 0.001, **(D**_**3**_**)** *p < 0.05 (Kruskal-Wallis test). *p*-values from Dunn’s *post hoc* test with Holm-Bonferroni correction are indicated by brackets with asterisks in the plot. The animal-based comparison revealed that the fraction of SLEs was reduced in 120-μmol/L-CNB cohort in comparison to the Crtl cohort (*p* = 0.0042), the SLE duration was shortened (Crtl vs. 120 CNB, *p* = 0.0049), and SLE rate was elevated in the 60-μmol/L-CNB group compared to the Ctrl group (*p* = 0.015).

**Figure 2:**
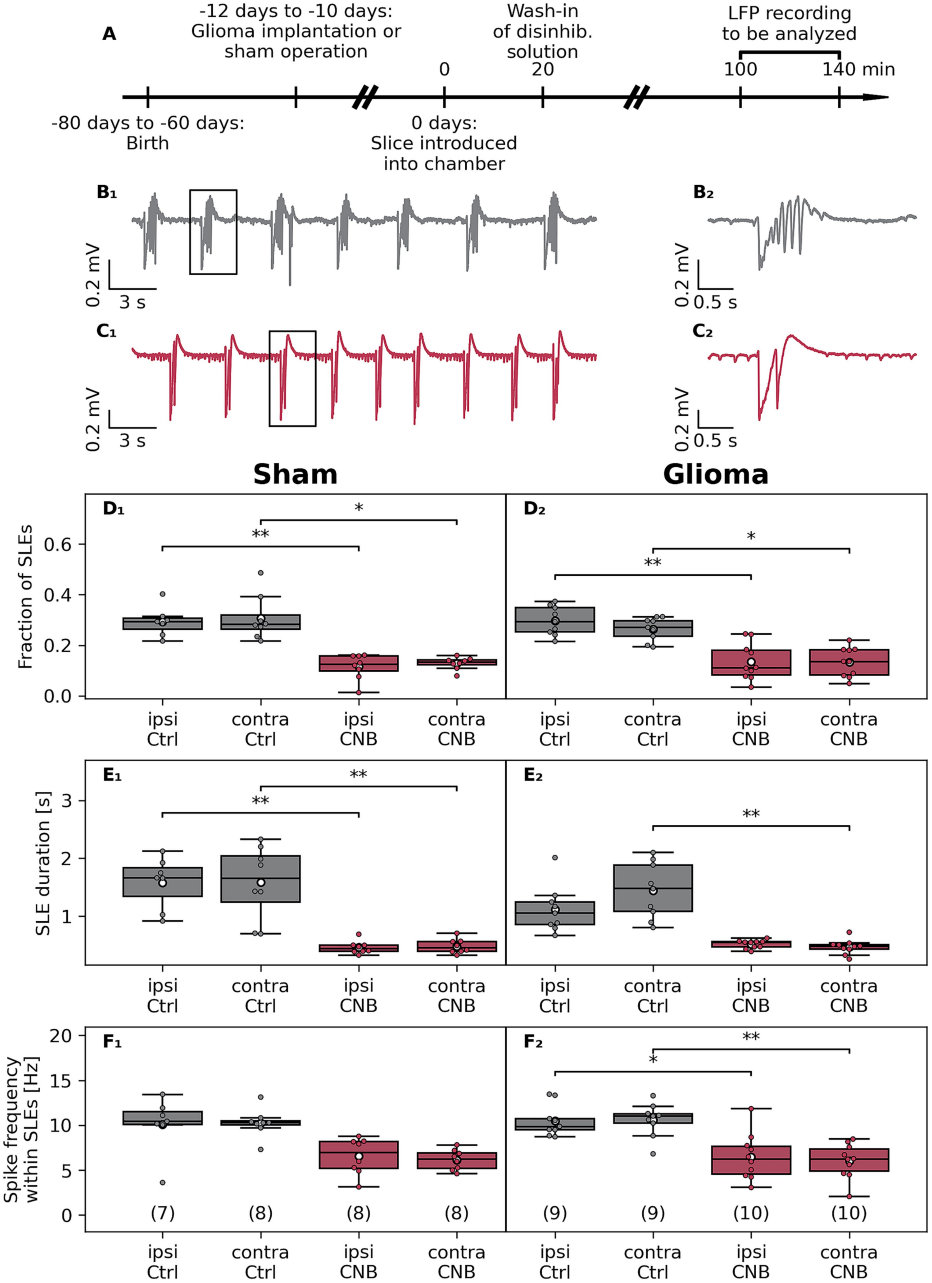
Effects of cenobamate on *ex vivo* network excitability in the F98 glioma Fischer rat model. **(A)** Male Fischer rats at the age of 50–70 days were implanted with a glioma or underwent sham surgery. Animals were sacrificed after 10–12 days, coronal brain slices were obtained, and were subjected to electrophysiological investigation. **(B**_**1**_**)** Sample traces of local field potential (LFP) recordings of a vehicle-treated (Ctrl) and cenobamate-exposed (120 μmol/L, **C**_**1**_) glioma-bearing slices. **(B**_**2**_**)** respectively **(C**_**2**_**)** represent magnified views of a seizure-like event (SLE) marked with a black box in **(B**_**1**_**,C**_**1**_**)**. **(D**_**1**_**–F**_**2**_**)** Quantitative analysis of SLE characteristics represented as boxplots. The number of coronal slices of each experimental group is given in brackets. In the sham-operated rat groups, slices were derived from 6, 7, 5, or 5 animals, respectively. All groups in the glioma-implanted cohort consisted of 7 rats. Individual data points are presented as full-colored dots. Median is shown as a black-colored line, and the mean is illustrated as a white-colored circle; **(D–F)** *p < 0.05, **p < 0.01,***p < 0.001 (Kruskal-Wallis test). *p*-values from Dunn’s post hoc test with Holm-Bonferroni correction are indicated by brackets with asterisks in the plot. Statistical tests were applied to data in each row, considering not only CNB treatment but possible effects of the glioma as well. Analyzing the data in an animal-based manner revealed similar results as in the slice-based analysis, but in some cases failed to reach a significant level due to the limited number of data points. Within the Sham group, a reduced fraction in both ipsilateral (ipsi Ctrl vs. ipsi CNB, *p* = 0.032) and contralateral hemispheres (contra Ctrl vs. contra CNB, *p* = 0.042) was determined after CNB exposure, which also was seen in part in the glioma groups: ipsi Ctrl vs. ipsi CNB (*p* = 0.041). The duration of SLE in sham-operated slices was reduced: ipsi Ctrl vs. ipsi CNB (*p* = 0.019), contra Ctrl vs. contra CNB (*p* = 0.034). Like in the slice-based calculation, duration in slices from glioma bearing animals was reduced in the contralateral hemisphere (contra Ctrl vs. contra CNB, *p* = 0.021). The spike frequency within SLEs was unaffected by an application of CNB.

The experimental timeline for naive rats is shown in Figure 1A; the timeline for glioma-bearing and sham-operated animals is illustrated in Figure 2A. We recorded LFPs to evaluate the effect of CNB at 60 and 120 μmol/L in both disinhibition models (Supplementary Figure 1; Figure 1). These comparisons led us to select the disinhibition model (II), containing additional 50 μmol/L 4-AP for all further experiments of the study. The first disinhibition model did not always yield clearly recognizable SLE onsets and terminations. Additionally, compared to initial recordings based on the 4-AP disinhibition model, the electrolyte-based model w/o 4-AP exhibited significantly more pauses in seizure-like activity lasting longer than 2 min (model (I): 10/18 slices vs. model (II): 1/27 slices; Fisher exact test: *p* < 0.001; Supplementary Figure 2).

### 2.5 Identification and characterization of seizure-like event metrics

We quantified the proportion of recording time occupied by SLEs (referred to as SLE fraction), SLE duration, spike frequency within SLEs, and SLE rate (Supplementary Figure 3). We employed a WaveNet-based deep learning model (Géron, 2019; van den Oord et al., 2016) to delineate SLE onsets and terminations. The model was trained on 30-s segments of human-labeled recordings, downsampled to 125 Hz. Each data point of these segments was labeled as part of an SLE or not. Deep learning was implemented in Python using Keras (3.3.3)/TensorFlow (2.16.1). SLEs, predicted by the model, which were shorter than 0.1 s were omitted, as those most often were interictal events or noise which the model had misidentified to be SLEs. SLEs with an SLE interval of less than 0.2 s were regarded as one SLE. All SLE endpoints were then algorithmically adjusted to align with their respective final extremum (final minimum or maximum, Supplementary Algorithm 1).

The model was used to sequentially label all recordings. We manually reviewed the algorithmically corrected outputs every 3–6 recordings using a custom Python script with an HTML and JavaScript-based frontend for each recording, allowing for manual correction of labels when necessary. We then included these recordings and their labels in the training data. The small model architecture allowed for rapid retraining with the manually corrected batch of recordings. This progressively reduced the amount of human intervention required for each batch of recordings. Training data were augmented by duplicating the original dataset and inverting the sign of the duplicated half. Synthetic noise, scaled to mimic the characteristics observed in actual recordings, was also added. Specifically, 25% of the training data remained unchanged; 25% had an additional, weak, approximately 50 Hz signal mixed with Gaussian noise; 25% had 5-s portions within each 30-s segment replaced by short bursts of Gaussian noise; and the remaining 25% had random sample points replaced with values drawn from a Gaussian distribution. Each 30-s segment was standardized by subtracting its mean and dividing by its standard deviation. Training and validation data were split such that each recording was exclusively assigned to either the training set or the validation set. This was necessary because SLE morphologies within each recording tended to be very similar. The total duration occupied by SLEs and the proportion of CNB-treated recordings were balanced between training and validation sets (Supplementary Table 1). The final model was trained for 100 epochs on approximately 3,500 min of data and validated on 860 min using the binary cross-entropy loss function, achieving an algorithmically corrected (as described above) validation accuracy of 97.4% (Supplementary Table 2). If no improvement in validation accuracy was observed for more than 2 epochs, the learning rate was divided by 10.

Within each event, spikes were detected by identifying local minima using SciPy’s argrelmin function. The order parameter was set so that each data point was evaluated within a 0.165-s window before and after to determine the presence of a local minimum.

We performed a slice-based analysis (2–6 slices from one animal in an experimental group), in which each slice was counted as one data point. In addition, the data were utilized in an animal-based manner (all *ex vivo*-stimulated slices of one experimental group obtained from one animal were merged).

### 2.6 Analyzing the power spectral density of whole recordings

For the final 40 min of each recording, the power spectral density (PSD) was computed using SciPy’s periodogram function with a Hann window and scaling was set to “density.” Frequency components below or equal to 0 Hz and above 30 Hz were removed, and the remaining PSD values were normalized by dividing each value by the total power within this frequency range. The normalized PSD was then binned into 0.2 Hz intervals. Bootstrapping generated a bootstrap distribution by repeatedly sampling with replacement from the observed distribution of PSDs (considering each recording as one sample). Confidence intervals were subsequently determined from this bootstrap distribution. The sampling process was repeated 10^4^ times.

### 2.7 Data analysis and statistical analysis

Data analysis was performed using custom Python (3.11.8 for deep learning and 3.12.3 for all other purposes) scripts. *Post hoc* statistical tests were conducted in Python and custom graphical user interfaces (GUIs) were implemented either via Tkinter (8.6.13), Flask (3.1.0), HTML5 and JavaScript (≥ES6/ES2015). Statistical analyses were conducted using custom Python scripts, primarily utilizing the SciPy (1.13.1) and scikit-posthocs (0.7.0) libraries. The Fisher exact test was used to test whether the purely electrolyte-based disinhibition model showed more pauses in seizure-like activity. Mean group differences were tested for significance using the nonparametric Kruskal–Wallis test before, for multiple comparisons, subgroups were tested with the *post hoc* Dunn’s test. Post hoc test results and Fisher-exact test results are indicated as follows: *p < 0.05, **p < 0.01, ***p < 0.001.

## 3 Results

### 3.1 Cenobamate lowers epileptiform metrics in slices from naive animals

The overall aim of the study was to analyze the effect of CNB on a preclinical glioma model, which is associated with tumor-related seizures (Bouckaert et al., 2020; Lange et al., 2021). To this end, coronal slices of naive animals were challenged with two doses of CNB, and seizure-like events (SLE) were analyzed (Figure 1), Kruskal-Wallis test, followed by *post hoc* Dunn’s test. CNB doses used in this *ex vivo* study mirror clinically relevant plasma levels used in the treatment of focal epilepsy (Steinhoff et al., 2024). CNB significantly reduced the SLE fraction (relative time occupied by SLEs) to 63% (60 μmol/L CNB, *p* < 0.01) and to 44% (120 μmol/L CNB, *p* < 0.001, Figure 1D_1_). Additionally, the duration of SLEs was shortened after anticonvulsant exposure to 32% (60 μmol/L CNB) and 27% (120 μmol/L CNB, for both *p* < 0.001, Figure 1D_2_), respectively. As illustrated in Figure 1D_3_, spike frequency within SLEs was attenuated by the high dose of CNB to 79% (*p* < 0.05), an effect not observed at 60 μmol/L. In contrast to the diminishing impact of CNB on the fraction, duration, and spike frequency within an event, the SLE rate was determined to be elevated by anticonvulsant exposure (Figure 1D_4_). In comparison to vehicle-treated controls, SLE rate was increased to 191% (*p* < 0.001) and 151% (*p* < 0.05), respectively, after treatment with 60 or 120 μmol/L CNB.

### 3.2 Cenobamate lowers epileptiform metrics in slices from glioma-bearing animals

Since we found a hyperexcitability-suppressing effect of CNB in naive rats, the experiments were extended to the syngeneic Fischer/F98 glioma model (Figure 2). Here, we focused on experiments using only 120 μmol/L CNB. In slices from sham-operated and glioma-bearing rats, CNB reduced the fraction of time occupied by SLEs in all groups (Figures 2D_1_,D_2_). In controls, the fraction of SLEs was reduced to 39% (*p* < 0.01) and 42% (*p* < 0.05) in the ipsilateral and contralateral hemispheres, respectively. Similarly, in glioma-bearing slices, the proportion of SLE was reduced to 45% (ipsilateral, *p* < 0.01) and 50% (contralateral, *p* < 0.05) by CNB incubation.

Consistent with our findings in naive animals, incubation in CNB significantly reduced SLE duration in slices of sham-operated animals (ipsilateral to 29%; contralateral to 30%; *p* < 0.01; Figure 2E_1_). In F98-bearing slices, CNB incubation similarly reduced SLE duration on the contralateral side to 32% (*p* < 0.01), although this reduction did not reach significance on the ipsilateral side (46%, *p* = 0.11, Figure 2E_2_).

Next, we investigated whether CNB incubation affected spike frequency within SLEs. As illustrated in Figure 2F_1_, no significant reduction was found in sham-operated animals (ipsilateral: 65%, *p* = 0.17, contralateral: 60%, *p* = 0.056). However, in tumor-bearing slices, spike frequency was significantly reduced in ipsilateral (62%, *p* < 0.05) and contralateral (57%, *p* < 0.05) hemispheres (Figure 2F_2_). Regarding SLE rate, no significant differences in both experimental cohorts were determined (Supplementary Figure 4).

### 3.3 Power spectral density analyses confirm dampening effects of cenobamate on epileptiform activity

To verify whether our previous analyses based on SLEs might have missed critical frequency components, we conducted power spectral density (PSD) analyses. Slices were not differentiated according to their ipsilateral or contralateral origin but were classified based on the animals’ experimental conditions: naive, sham-operated, or glioma-implanted. Additionally, slices were categorized according to treatment status: vehicle control or 120 μmol/L CNB.

**Figure 3:**
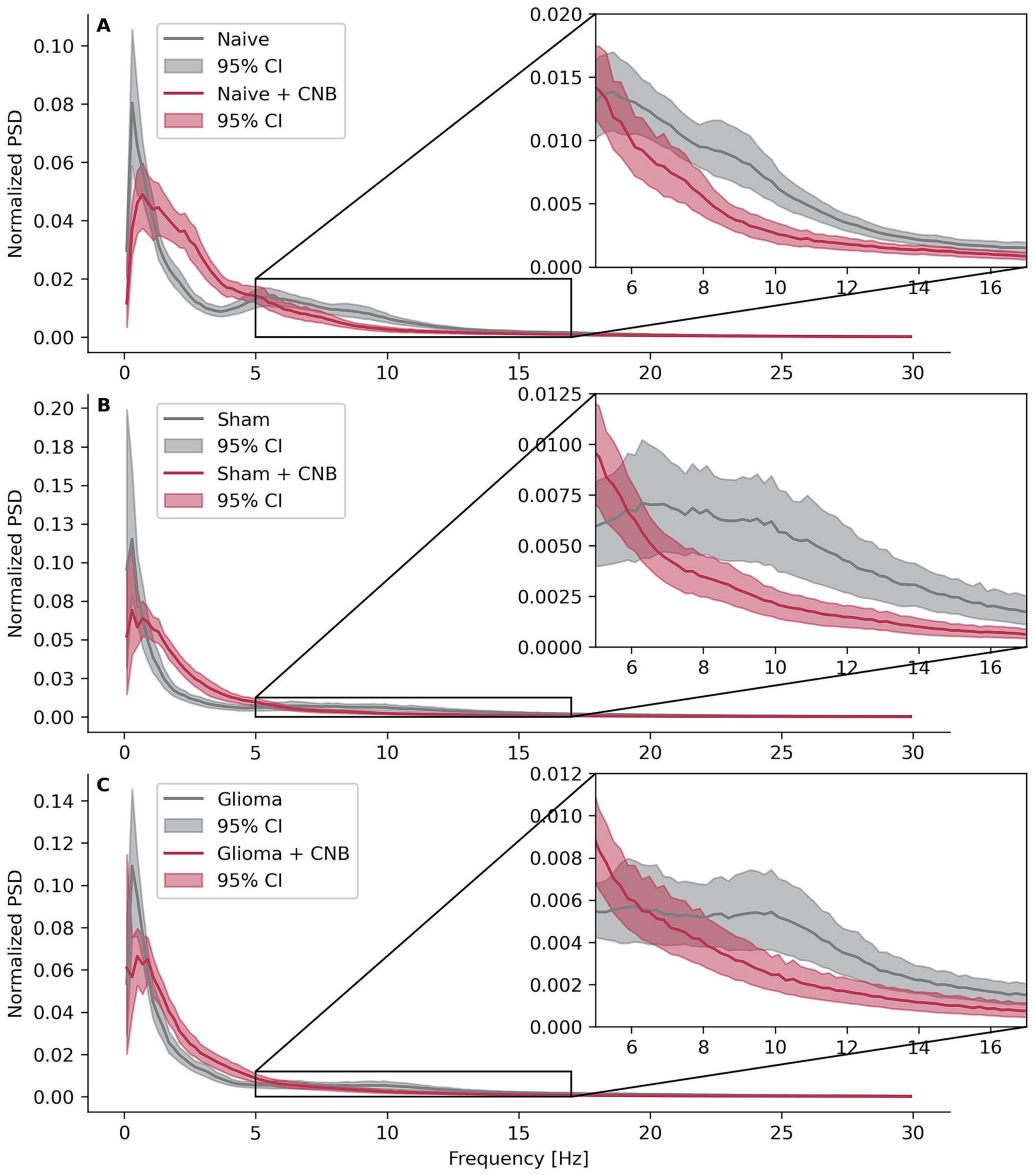
Power spectral densities. Effects of cenobamate on binned and normalized power spectral densities (PSD) of LFP recordings across the frequency 0 < × ≤ 30 Hz for different experimental groups. PSD values were normalized within the frequency range by dividing each data point by the sum of all points in that range and binned with a width of 0.2 Hz. Confidence intervals were bootstrapped. **(A)** Depicts calculations of naive animals, **(B)** sham-operated rats, and **(C)** rats implanted with F98 glioma.

All three groups, not treated with CNB, exhibited at least two distinct peaks in the normalized, 0.2-Hz-binned PSD. The first peak appeared below 1 Hz, corresponding to the presence of SLEs, while the second peak ranged between 6 and 14 Hz, aligning with the spike frequency typically observed during SLEs (approximately 10 Hz). In contrast, slices treated with CNB showed only a single peak below 1 Hz, and the prominent 10 Hz peak was absent. Furthermore, at ~10 Hz, the mean normalized PSD values for control slices consistently surpassed those of treated slices, with non-overlapping bootstrapped 95% confidence intervals (Figures 3A–C). Interestingly, the non-normalized PSD with 0.2 Hz binning showed higher values between 1 and 5 Hz, with non-overlapping bootstrapped confidence intervals (Supplementary Figure 5).

## 4 Discussion

**Figure 4:**
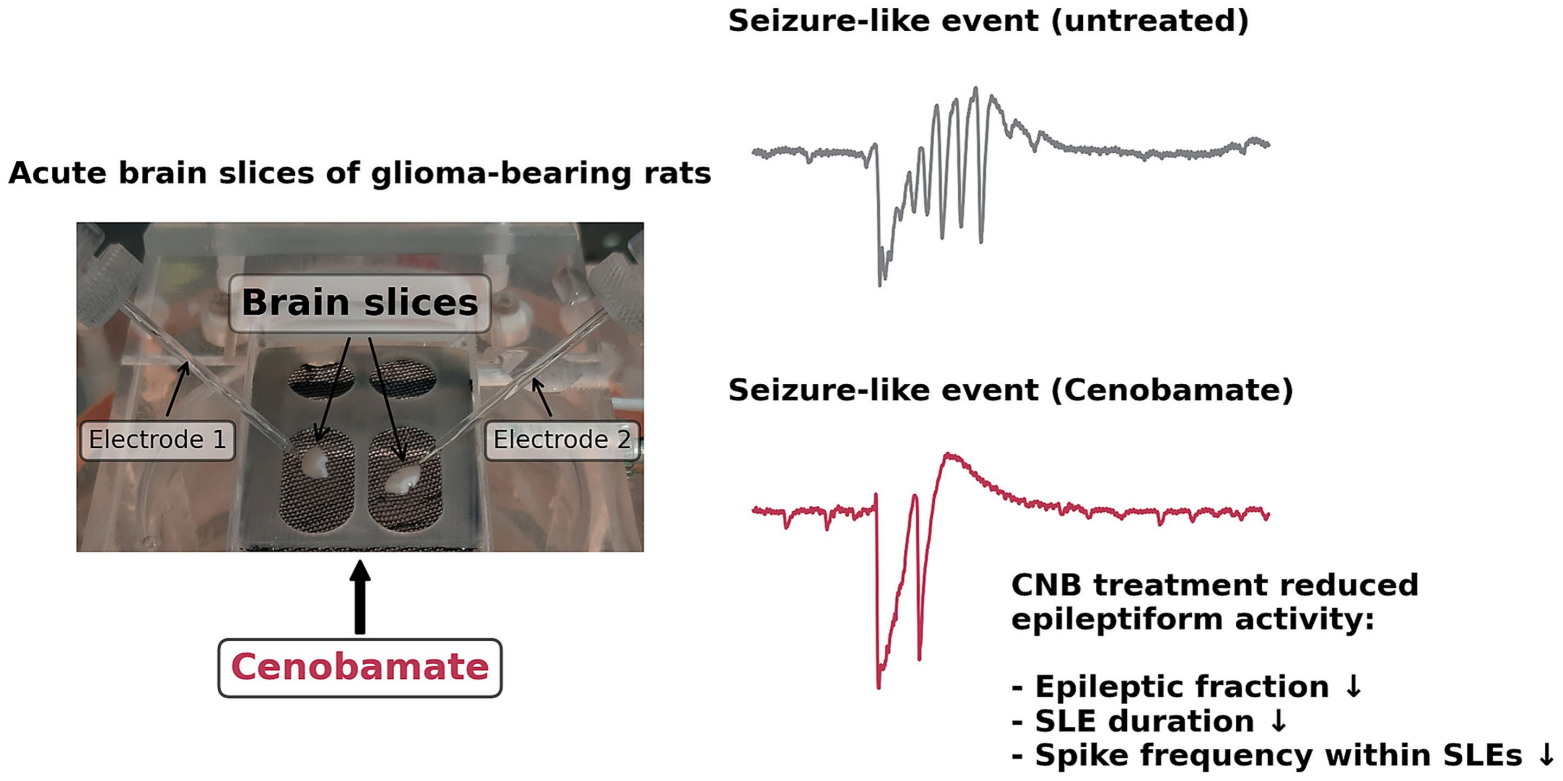
Schematic presentation of *ex vivo* cenobamate action in F98 glioma. Acute brain slices were challenged with clinically relevant doses of CNB, and seizure-like events were identified employing field potential recordings. Exposure to CNB led to a reduced fraction and duration of seizure-like events. In slices containing F98 tumors, spike frequency within events was also found to be reduced by exposure to CNB.

In the current management of glioma-associated epilepsy, achieving seizure-free conditions remains challenging. This study presents the first experimental investigation of the effects of cenobamate on glioma-bearing brain tissue. Under disinhibitory conditions (8 mM KCl, 0 mM MgCl_2_, 50 μmol/L AP), slices from glioma-bearing or sham-operated animals showed spontaneous groups of spikes, which we termed seizure-like events (SLEs). On the whole, we obtained CNB effects that were similar between glioma and sham tissue. To sum up the effects of CNB, our main findings are illustrated in Figure 4.

Our main finding of the present study was that CNB reduced the cumulative time spent with SLEs at concentrations comparable to those observed in human plasma (Steinhoff et al., 2024). Since this effect was found in both glioma and sham tissue, our data suggest that CNB suppresses neuronal hyperexcitability and that this effect is preserved in glioma-bearing tissue. The reduced SLE fraction was primarily due to a reduced SLE duration rather than a reduced SLE rate. This is consistent with the established mechanism of action of CNB, namely the preferential binding to the inactivated state of the voltage-gated sodium channel, thereby suppressing the non-inactivating (persistent) component of sodium currents (Nakamura et al., 2019). This shortens the sodium-induced depolarization and may explain the reduced SLE duration.

By definition, SLEs are composed of individual spikes. Since the spike rate could be independent of the SLE duration, we next analyzed spike frequency within SLEs. We found that spike frequency dropped significantly with CNB, but without a difference between glioma and sham tissue. Assuming that neuronal bursting may underlie spikes within an SLE, our data are consistent with the previous data that CNB accelerates the fast inactivation during sustained membrane depolarization and prolonged recovery time (Nakamura et al., 2019). Taken together, our findings may well be explained by the proposed mechanism of action of CNB.

As stated above, CNB did not decrease the SLE rate in our study, suggesting it may not block SLE initiation. In fact, we initially expected a drop in SLE rate because CNB was reported to act as a positive allosteric modulator of GABA_A_ receptors, enhancing tonic inhibitory currents (Sharma et al., 2020). It is important to note that CNB did reduce the SLE rate in the disinhibition model (I) without 4-AP (Supplementary Figure 1). Albeit we cannot fully explain this discrepancy, our data point to the possibility of some interference between CNB and 4-AP. At least there is some evidence that a pure electrolyte-based model primarily enhanced excitatory and glutamatergic activity (Mody et al., 1987), while the addition of 4-AP further disinhibited excitatory neurons and GABAergic inhibitory neurons (Codadu et al., 2019; Lado et al., 2022). Thus, the effect of CNB on SLE rate may be subject to the model used. Nonetheless, the SLE fraction, which was significantly reduced, is a product of SLE rate and SLE duration. So, if at all, the SLE rate was increased, SLE shortening clearly outranked this effect.

Are our CNB data comparable to data from other sodium channel blockers? One study used 0 mM magnesium to evoke events named “late recurrent discharges” (Zhang et al., 1995). The authors showed that phenytoin (50 μmol/L) and carbamazepine (50 μmol/L) increased the rate but decreased the duration of these events, similar to our findings. In contrast, the same study found that phenobarbital (150 μmol/L) had no significant effect on frequency but only slightly reduced event duration, while the purely GABAergic agent midazolam (50 μmol/L) had no significant impact on either of these measures. We hence conclude that our observed effects of CNB on spontaneous activity were primarily due to the acceleration of fast inactivation of sodium currents as well as to suppression of persistent sodium currents.

One might hypothesize that CNB treatment affects glioma progression via GABAergic mechanisms. However, the exact role of GABA in glioma progression is poorly understood. High expression of specific GABA receptor subunits has been correlated with improved overall survival, whereas overexpression of other subunits was associated with reduced survival (Badalotti et al., 2024; Wang et al., 2021). In interneurons, elevated GABAergic activity had been linked to reduced proliferation of tumor cells in a murine high-grade glioma model (Tantillo et al., 2020); conversely, tumor-promoting GABAergic neuron-to-glioma synapses had been observed in a patient-derived orthotopic xenograft model of diffuse midline glioma (Barron et al., 2025). Valproic acid, which was found to increase GABA levels (Romoli et al., 2019), had not been shown to decrease overall survival (Happold et al., 2016; Kuo et al., 2020). Furthermore, gabapentinoids, which elevate GABA concentrations (Cai et al., 2012), have even been shown to increase survival in glioblastoma patients (Bernstock et al., 2025), likely through a non-GABAergic mechanism. These findings might indicate that increased GABA-mediated receptor activation does not invariably lead to glioma progression.

We could not observe differences in SLE fraction, SLE duration, or SLE rate between glioma and sham tissue. In a previous study using gabazine-based disinhibition models, spontaneous events were rather less frequent in glioma-bearing tissue than in sham tissue (Lange et al., 2020). However, the 4-AP-based disinhibition model was associated with comparable event rates in both tissue groups (~12/min, Lange et al., 2020), which, in addition, was comparable to the SLE rate observed in the present study. It is thus of note that CNB could reliably and effectively attenuate hyperactivity in glioma-bearing tissue, i.e., with the same potency as in sham-operated or naive control tissue. What might be the clinical relevance? We suggest that CNB may be a valuable addition to the treatment toolkit for glioma-related epilepsy. Seizure management in patients with glioma may be complicated by potential drug interactions between antiepileptic drugs (AEDs) and chemotherapeutic agents. This is why CYP450 enzyme-inducing AEDs such as phenytoin and carbamazepine are generally avoided (van der Meer et al., 2021; Weller et al., 2014). Levetiracetam and valproic acid are currently among the most commonly prescribed first-line AEDs for glioma patients (Avila et al., 2024). However, valproic acid, a CYP450 inhibitor, may increase the toxicity of certain chemotherapeutic agents. In addition, common adverse effects such as thrombocytopenia, weight gain, and tremor can lead to discontinuation of treatment (van der Meer et al., 2021). Although levetiracetam is often well tolerated, psychiatric side effects such as agitation, fatigue, and somnolence may require a change of treatment in some patients (van der Meer et al., 2021; Yi et al., 2019). Since the correct dose titration of CNB often takes several weeks, the anticonvulsant may be particularly beneficial for patients suffering from low-grade glioma with pharmacoresistant seizures, as this cohort has a significantly longer overall survival time than patients diagnosed with glioblastoma.

In our study, we used male Fischer rats as hosts for orthotopic F98 glioma implantation. We cannot rule out that effects of CNB may differ in female animals. However, in clinical studies, no differences between both sexes in the anticonvulsant efficacy of CNB have been reported so far (Krauss et al., 2020; Klein et al., 2022; Krauss et al., 2024; Krauss et al., 2025).

In summary, in a rodent *ex vivo* model of glioma, we demonstrate for the first time that CNB exhibited diminishing effects on the hyperexcitability of peritumoral networks. Our data indicate reduced duration and decreased spike frequency of seizure-like events in all investigated groups. Since our study focused on *ex vivo* application, follow-up investigations of CNB action in glioma should be examined under *in vivo* conditions in animal models of tumor-associated epilepsy.

## Data Availability

The raw data supporting the conclusions of this article will be made available by the authors, without undue reservation.
