# Supplementary material for: Cenobamate reduces epileptiform activity in the ex vivo F98 rat glioma model
Source: Front Neurosci. 2025 Aug 5;19:1629259. doi: 10.3389/fnins.2025.1629259 (PMC12392110; doi:10.3389/fnins.2025.1629259)
Supplement: Supplementary file 1 [file Data_Sheet_1.pdf]

Supplementary Material

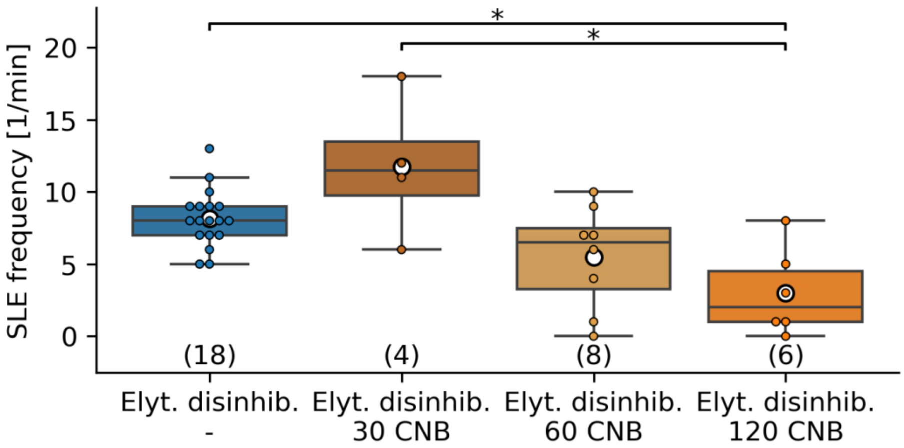

Supplementary Figure 1. Dose-response relationship of CNB on SLE rate in naive brain slices. Data were obtained from experiments employing disinhibition model I (w/o 4-AP). The number of slices per group is shown under the respective boxplot. Slices were established from the following number of rats: 5 (w/o CNB), 2 (30 µmol/L CNB), 3 (60 µmol/L CNB), and 3 (120 µmol/L CNB). The concentration of CNB is given in µmol/L. The mean of each group is marked with a white dot, the median is given by the black line. Individual data points are presented as full-colored dots. **$p<0.01$ (Kruskal-Wallis test), P-values from Dunn's post hoc test with Holm-Bonferroni correction are indicated by brackets with asterisks in the plot.

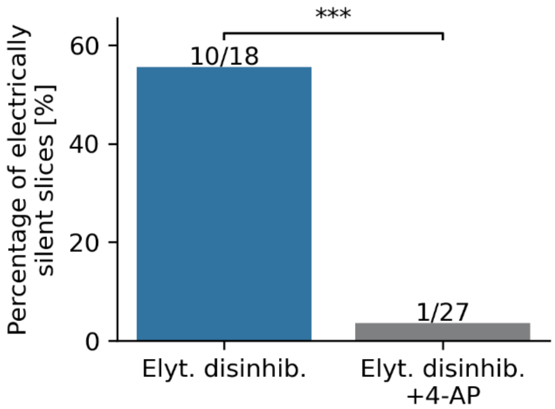

Supplementary Figure 2. Barplot indicating the percentage of slices showing electrical silence for at least 2 continuous minutes. ***$p<0.001$ (Fisher-exact test).

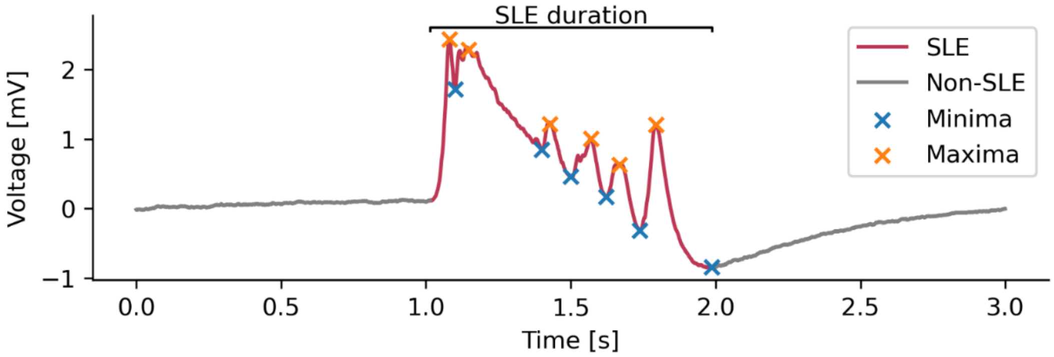

Supplementary Figure 3. Exemplary seizure-like event (SLE) highlighting how field potential deflections were analyzed. Segments identified algorithmically as part of an SLE are highlighted in red, whereas interictal phases (marked as Non-SLE) are shown in grey. The last extremum within each SLE was always considered as endpoint of that event. Minima and maxima are marked with '×'. These local extrema were detected using SciPy's argrelmin and argrelmax function. The order parameter was set so that each data point was evaluated within a 0.165-second window before and after to determine the presence of a local minimum or maximum.

The following pipeline was used to improve upon the output of the deep learning model.

Input:

- Predicted labels (from model)
- Time-series data (the recordings)

Output:

- Refined SLE label array

Pipeline

1. **Up sample** labels to match the 2000 Hz sampling rate of original recordings.
2. **Merge** all SLEs that are too close to each other.
3. **Remove** any SLEs that are too short to qualify as true events.
4. **For each remaining event** do:
   4.1. **Obtain starts and ends** based on the predicted labels.
   4.2. **Center the SLE** by subtracting the Mean of the non-SLE parts of the recording.
   4.3. **Determine polarity** of the SLE ending by summing signal values within ±0.015 seconds around the event's last time point.
   4.4. **if** the ending sum is positive **then**
      4.4.1. **Find the last local minimum** of the cumulative sum (scipy.signal.argrelmin, order = 25).
      4.4.2. **Locate the last maximum** within 0.2 seconds after that local minimum. This is the new endpoint.
   4.5. **else if** the ending sum is negative **then**
      4.5.1. **Find the last local maximum** of the cumulative sum (scipy.signal.argrelmax, order = 25). This is the new endpoint.
      4.5.2. **Locate the last minimum** within 0.2 seconds after that local maximum.
   4.6. **end if**
5. **Return** the refined label array containing merged and corrected endpoints.

Supplementary Algorithm 1. Simplified algorithm describing the refinement process for model predictions, primarily aimed at ensuring a consistent definition of seizure-like event (SLE) termination and eliminating misclassification of short interictal spikes as SLEs.

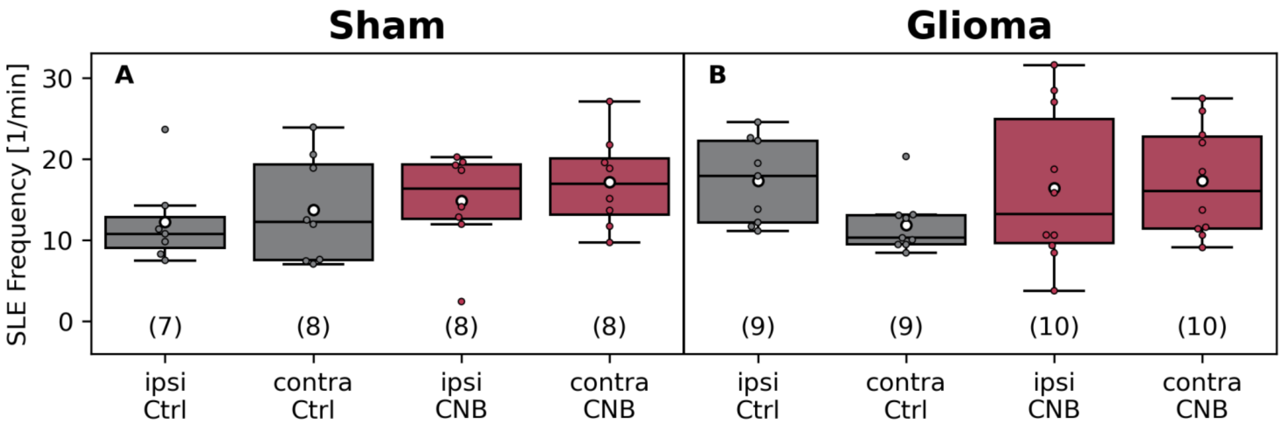

Supplementary Figure 4. **Analysis of SLE rates in slices from glioma-bearing Fischer 344 rats**. (**A**) shows the effect of 120 µmol/L CNB treatment on SLE rate for slices from sham-operated animals. (**B**) shows the effect on glioma-bearing animals. The mean of each group is marked with a white dot, the median is given by the black-colored line. Individual data points are illustrated as full-colored dots. No significant differences were identified. The number of coronal slices is given in brackets. In the cohort of sham-operated animals, brain slices were derived from the following number of rats: 6 ipsilateral Ctrl, 7 contralateral Ctrl, 5 ipsilateral CNB, and 5 contralateral CNB. Slices of the F98 glioma-bearing cohort were established from 7 rats in each experimental group.

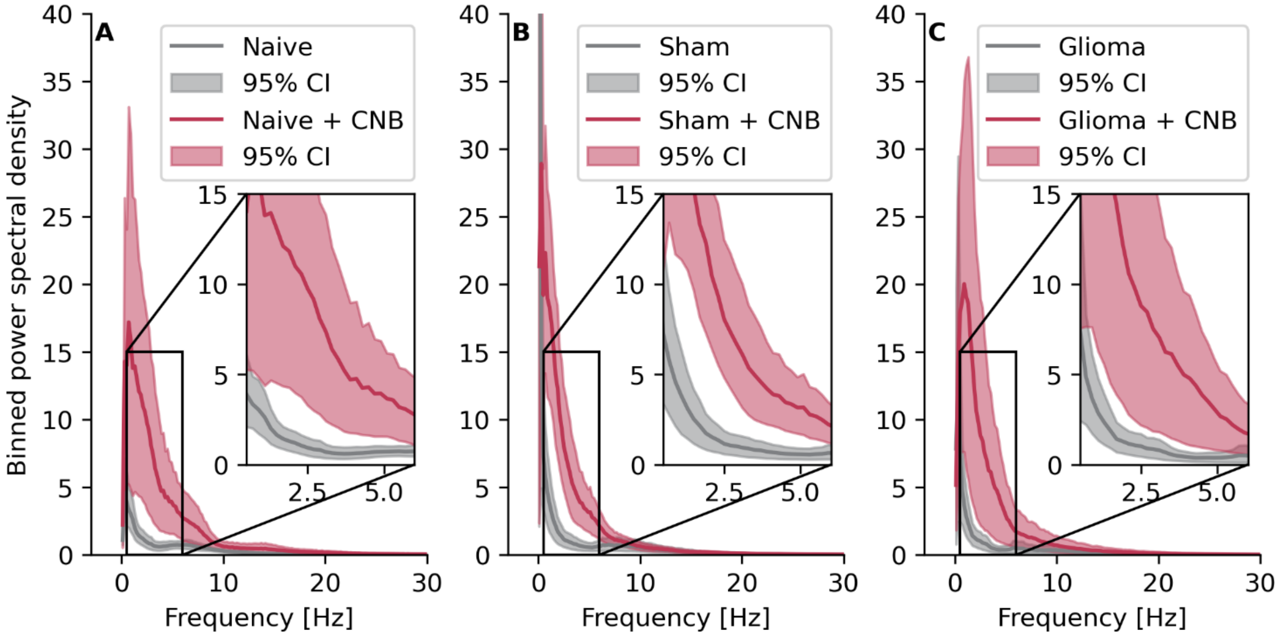

Supplementary Figure 5. **Power spectral densities (PSDs) of local field potential (LFP) recordings across the $0 < X \leq 30$ Hz frequency range for different experimental groups**. Data were segmented into 0.2 Hz bins but not normalized. Notably, PSD values between approximately 1 and 5 Hz are increased under 120 µmol/L CNB treatment across all groups. All confidence intervals were estimated using bootstrapping. (**A**) naive animals, (**B**) sham-operated rats, and (**C**) F98 glioma-bearing rats.

Our WaveNet implementation builds upon the architecture and code presented in Chapter 15 of Hands-On Machine Learning with Scikit-Learn, Keras & TensorFlow by Aurélien Géron (2nd Edition. O'Reilly Media, 2019) and Oord et al. (2016). However, our model utilizes a single block composed of 10 convolutional layers, each containing gated activation units with 28 filters. Our model produces a 256-dimensional output, followed by a final Dense layer with a sigmoid activation function, reducing the output shape to the input shape.

Supplementary Table 1: **Training and validation data used in the final training of the WaveNet model.** Slices from the same animal could be included in both datasets; however, snippets from an individual recording were never shared between training and validation sets. Data were further stratified based on CNB treatment and the proportion of time occupied by SLEs.

	**Training data**	**Validation Data**
**n recordings**	88	22
**n animals**	22	16
**n recordings CNB treated**	40 (45%)	10 (45%)
**total recording time**	3502.5 minutes	862.5 minutes
**time occupied by SLEs**	29.99%	28.87%

Supplementary Table 2: Results from training the final model on the complete dataset. Although the validation accuracy only improved slightly with algorithmic correction, this modest improvement does not reflect its importance. Without the removal of raw predictions that occasionally mislabeled brief interictal spikes as SLEs, the averages of the SLE-related metrics may have been strongly distorted.

	**Epoch**	**Validation results**			**Algorithmically improved validation results**		
		Prec.	Rec.	Acc.	Prec.	Rec.	Acc.
**Results from the epoch with best pure validation results**	32	0.959920	**0.896293**	**0.968382**	0.951321	**0.930932**	0.973855
**Results from epoch with best algorithmically improved prediction on the validation data**	26	**0.960181**	0.895850	0.968344	**0.951701**	0.930745	**0.973903**
